# Study protocol to assess de-implementation of the initial provider encounter for diagnosis and treatment of obstructive sleep apnea: the DREAM (Direct Referral for Apnea Monitoring) Project

**DOI:** 10.1186/s12890-022-01899-y

**Published:** 2022-04-02

**Authors:** Robert L. Folmer, Eilis A. Boudreau, Charles W. Atwood, Connor J. Smith, Annette M. Totten, Jamie L. Tock, Priyanka Chilakamarri, Kathleen F. Sarmiento

**Affiliations:** 1 VA Portland Healthcare System, Portland, OR USA; 2 Department of Otolaryngology, Oregon Health & Science University, Portland, OR USA; 3 Department of Medical Informatics and Clinical Epidemiology, Oregon Health & Science University, Portland, OR USA; 4 Department of Neurology, Oregon Health & Science University, Portland, OR USA; 5 VA Pittsburgh Healthcare System, Pittsburgh, PA USA; 6 San Francisco VA Healthcare System, San Francisco, CA USA; 7 University of California, San Francisco, CA USA; 8 National Center for Rehabilitative Auditory Research (NCRAR), VA Portland Medical Center, Portland, OR 97239 USA

**Keywords:** Obstructive sleep apnea, Home sleep apnea testing, Polysomnography, Negative predictive value

## Abstract

**Background:**

Obstructive sleep apnea (OSA) is a very common and serious health condition which is highly prevalent among U.S. military Veterans. Because the demand for sleep medicine services often overwhelms the availability of such services, it is necessary to streamline diagnosis and treatment protocols. The goals of this study are to, (1) assess the efficacy of de-implementing the initial provider encounter for diagnosis and treatment of OSA; (2) determine the negative predictive value (NPV) of home sleep apnea testing (HSAT); (3) develop HSAT usage recommendations for various at-risk patient populations.

**Methods:**

This is a large, pragmatic study that will take place in 3 VA sleep medicine programs: San Francisco, CA; Portland, OR; and Pittsburgh, PA. All Veterans referred for new sleep apnea evaluations at these sites will be included in this four-year study. Outcomes will include time from referral for OSA to sleep testing and treatment; positive airway pressure (PAP) treatment adherence measures; patient-reported clinical outcomes and measures of satisfaction; determination of the NPV of HSAT; HSAT usage recommendations for at-risk patient populations.

**Discussion:**

The DREAM (Direct Referral for Apnea Monitoring) Project will inform sleep medicine providers and clinical organizations regarding strategies to streamline diagnosis and treatment protocols for OSA. Results of this study should have significant impact on clinical practices and professional guidelines.

*Trial registration* The majority of this project is an observational study of clinical procedures. Therefore, clinical trial registration is not required.

**Supplementary Information:**

The online version contains supplementary material available at 10.1186/s12890-022-01899-y.

## Background

More than 10% of the general population and 30% of the U.S. Veteran population are at risk for obstructive sleep apnea (OSA) [1]. OSA is characterized by repeated episodes of breathing cessation throughout the night and is associated with poor daytime functioning, increased risk of accidents, poor occupational performance, disruption of interpersonal relationships, and overall reduced quality of life [2]. Untreated OSA is an independent risk factor for developing hypertension, diabetes mellitus, stroke, and heart disease [3, 4]. However, treatments currently available can reverse the daytime impairment and long-term complications associated with sleep apnea [5]. While approximately 80% of individuals suspected of having OSA remain undiagnosed [6, 7], the rates of referral and diagnostic testing continue to rise each year, placing considerable strain on limited resources, especially in closed health care systems such as the Veterans Health Administration (VHA) [8, 9]. Therefore, identifying and addressing system inefficiencies and barriers is critical to providing patients with improved access to care.

In 2020, the U.S. Department of Veterans Affairs (VA) Health Services Research & Development (HSR&D) division funded a 4-year study (award #IIR 15-339) to evaluate barriers that limit the detection and treatment of OSA, including:

1. The necessity of an initial provider encounter for newly-referred patients.
  Currently, Medicare and most insurance companies require a physician or licensed independent provider visit prior to sleep testing and/or initiation of therapy. At some VA facilities, the wait time to see a sleep provider can be up to 6 months, and in underserved areas community care may not be available or may have even longer wait times than VA [10]. Therefore, a key question is whether de-implementation of the initial sleep provider visit via substitution with an electronic chart review or e-consult improves timeliness to diagnostic/therapeutic care while preserving care integrity and effectiveness.
2. The use of home sleep apnea testing (HSAT) in a broad array of comorbid conditions, regardless of pretest probability of disease, and focused specifically on assessing the negative predictive value of HSAT.
  The second barrier this study addresses is whether HSAT can be used in patients with significant co-morbidities. In recent years, HSAT—which is less expensive than in-lab polysomnography (PSG) and can be conducted in the patient’s home—has gained wide-spread acceptance. However, the effectiveness of HSAT in identifying OSA has only been demonstrated in individuals who lack major co-morbidities, and is only validated to rule in sleep apnea. Also, the negative predictive value (NPV) of HSAT is not known, resulting in the need to confirm negative HSATs with more expensive in-lab testing which often has limited availability.

VA is uniquely positioned to address both the efficacy of e-consultation (in place of an initial provider encounter) and the negative predictive value of HSAT because VHA has a large population of Veterans at increased risk for OSA who also exhibit substantial co-morbidities [11]. Furthermore, the VA healthcare model emphasizes high quality, high-throughput and timely access to care for Veterans. This article describes the study protocol, its rationale, and plans for implementation, data collection and analysis.

## Study rationale

### Role of the initial sleep provider visit

The value of an initial sleep specialty care visit *before testing* for apnea has not been studied. Therefore, it remains unknown whether seeing a sleep specialist prior to testing impacts how patients engage in their care (e.g., keeping testing and clinic appointments, using therapy when prescribed, and understanding how OSA impacts their health). We suspect that the value of a sleep specialist visit may be more important for long-term chronic disease management rather than initial risk-stratification of patients for OSA. To this end, there have also been no studies that evaluated de-implementation of the initial sleep specialist visit for new OSA referrals on metrics of Veterans’ access to care. In 2016, Baig and colleagues at the Milwaukee VA published a retrospective review that evaluated how triaging patients directly to sleep testing or to initiation of therapy impacted wait times for treatment [12]. Unfortunately, the only results published were the reduction of days before positive airway pressure (PAP) therapy was prescribed for patients with existing OSA. No data was provided on wait time reduction by scheduling patients directly for sleep testing. Nonetheless, the reduction in wait times for prescription of PAP therapy averaged 53 days (going from 60 to < 7 days). These data are informative as a potential strategy to reduce wait times for the substantial number of Veterans with known OSA who are referred to VA sleep programs.

The first two Specific Aims of the study are:

- *Aim 1* Compare the time from referral to sleep testing and treatment of OSA in Veterans in the Traditional clinical pathway, which includes an initial encounter with a care provider prior to testing vs. the DREAM (Direct Referral for Apnea Monitoring) pathway, which includes a medical chart review and triage, but no initial encounter with a care provider prior to testing.
- *Aim 2* Compare PAP treatment adherence and patient-reported clinical outcomes/satisfaction in Veterans who follow the Traditional or DREAM clinical pathways.

If the DREAM pathway in not inferior to the Traditional clinical pathway for new OSA patients, the protocol should help to reduce the time from referral to diagnosis and treatment. The DREAM procedure should also reduce some of the workload burden for sleep care providers, which will enable them to provide clinical services more efficiently.

### Negative predictive value of HSAT

The use of home sleep testing among Veterans has increased every year since 2000 and surpassed polysomnography (PSG) as the most common VHA testing method in 2018 [11]. Despite the high prevalence of OSA and a large number of comorbid conditions in affected patients, there have been no significant changes in clinical guidelines regarding the use of HSAT for the detection of OSA. Guidelines on the use of HSAT are woefully outdated due to a lack of evidence to support a change. For example, an American Academy of Sleep Medicine (AASM) Practice Guideline from 2017 states [13], “We recommend that PSG, rather than HSAT, be used for the diagnosis of OSA in patients with significant cardiorespiratory disease, potential respiratory muscle weakness due to neuromuscular condition, awake hypoventilation or suspicion of sleep related hypoventilation, chronic opioid medication use, history of stroke or severe insomnia.” Although the Guideline authors rated the strength of this recommendation as “Strong”, they rated the evidence quality for it as “Very Low.” The 2017 Practice Guideline [13] also states that the “vast majority of well-informed patients [in this group] would most likely choose PSG to diagnose suspected OSA” with no evidence cited to support the assertion. In fact, a study by Skomro et al. [14] reported that 76% of patients preferred home sleep testing for OSA over in-lab PSG. In 2018, AASM published a Position Statement [15] on clinical use of HSAT that stated, “the need for, and appropriateness of, an HSAT must be based on the patient’s medical history and a face-to-face examination by a medical provider, either in person or via telemedicine.” Again, no evidence was cited to support the recommendation for “face-to-face examination” of patients prior to home sleep testing for OSA.

HSAT technology monitors cardiopulmonary systems and/or arterial tonometry to establish the presence or absence of sleep-disordered breathing. The comparative gold standard for OSA diagnosis is in-laboratory PSG. Home testing technology has been in use for more than 20 years, and advancements have led to excellent detection and differentiation of obstructive apneas from central apneas (blockage in the airway versus no effort to breathe) [16]. It has been demonstrated in multiple studies that false positive HSAT results are rare due to their use of total recording time rather than sleep time (i.e., sleep time is not directly measured) [17]. Underestimation of breathing events during HSAT also contributes to the test’s low false positive rate [18]. HSAT respiratory events rely solely on oxygen desaturation of at least 3% to score a breathing event. To minimize false negative results with HSAT, only patients highly likely to desaturate or who are likely to have a high Apnea–Hypopnea Index (AHI) should be triaged to HSAT. Thus, the use of HSAT requires a high degree of clinical suspicion for OSA based on classic risk factors and those that will increase the likelihood of desaturation with breathing events (obesity in particular).

In 2017, Cairns et al. [19] published results of HSAT outcomes from 1500 Veterans and 1500 non-Veteran sleep clinic patients. Although Veterans were more likely to exhibit co-morbidities such as depression, insomnia, hypertension, diabetes, and restless legs syndrome, no significant differences between the groups were observed for rates of positive HSATs, study integrity indicators, or predictors of OSA. These results highlight the need for additional studies that will provide evidence to inform clinical guidelines regarding the use of HSAT.

Specific Aim 3 of this study will determine the negative predictive value of HSAT by comparing results of the home test with PSG results obtained from the same patients. Also, HSAT results from a large number of patients will be collected and correlated with demographic and comorbidity data. This information should help to inform clinicians and policymakers regarding HSAT usage guidelines for various at-risk patient populations.

## Methods

### Study settings

This is a large, pragmatic study that will take place in 3 VA sleep medicine programs: San Francisco, CA; Portland, OR; and Pittsburgh, PA. All patients referred for new sleep apnea evaluations at these sites and meet the inclusion criteria will be included in this 4-year study.

### Eligibility and waiver of consent

Eligibility will be determined at the time of receipt of the referral for each patient. Reasons for referral, listed on the consult, will identify patients who are new evaluations for sleep apnea testing. Chart reviews will be performed to ensure that the reasons for referral are correct. A waiver of consent was obtained from each site’s Research & Development (R&D) Committee for Specific Aims 1 and 2, given that the research involves no more than minimal risk to participants, the wavier does not adversely affect the rights and welfare of the participants, and this research could not practicably be carried out without the waiver of consent (due to adding a point of contact to the flow/clinic processes, causing delays in care). All patients referred to each site’s sleep program for OSA will be included in the study. Patients will be tracked by research staff at each study site. Written informed consent will be obtained from patients for Specific Aim 3, as described below.

### Power analysis and sample size

A power analysis was performed for the smallest expected effect for Specific Aim 1 using the statistical package *webpower* in R [20]. The sample size was computed based on an OLS (ordinary least squares) linear regression analysis with eight covariates, an expected small effect (*f*^2^ = 0.02), default critical value α = 0.05, and power β = 0.80. The results indicated that data from a minimum of 756 patients should be collected. Given that 10% of patients are expected to decline PAP treatment, an additional 76 patients’ data will be recorded, resulting in a target sample size of 832. Based on the project timeline of data collection over a period of three years, and the desire to maximize sample size for exploratory aims (machine learning algorithms), patient data will be collected beyond the minimum required sample size until the period for data collection has elapsed.

### Study procedures—traditional pathway: initial visit with sleep care provider

Medical records of patients referred to participating VA sleep clinics for OSA will be reviewed by a care provider. Each study site will follow its usual procedures to assign new OSA patients to the Traditional or DREAM clinical pathways. That is, patients will be triaged to one pathway or the other according to the clinical judgment of sleep care providers at each site. Veterans in the Traditional pathway may be scheduled for a visit with a care provider through in-person visits, telephone clinics, or video conferencing. Patients will be referred for either HSAT or PSG based on the clinical decision of the provider evaluating the patient. HSAT or PSG testing and interpretation will be conducted according to standard clinical practices at each study site. Baseline questionnaires will be completed by patients at the time of sleep testing (see the Data Collection section for a list of study questionnaires).

### Study procedures—DREAM pathway: direct-to-sleep testing

Medical records of patients referred to participating VA sleep clinics for OSA will be reviewed by a care provider. Patients in the DREAM pathway will be scheduled as recommended by the triaging clinician and will be referred for either HSAT or PSG based on the clinical decision of the provider evaluating the patient. The only VA contact patients will receive prior to sleep testing will be through the scheduler making the appointment for testing. HSAT or PSG testing and interpretation will be conducted according to standard clinical practices at each study site. Baseline questionnaires will be completed by patients at the time of sleep testing.

### Study procedures—treatment for OSA

Patients who test positive for OSA will be treated according to usual clinical practices at each study site. For example, Veterans diagnosed with sleep apnea (AHI ≥ 15 events/h or 5 ≤ AHI < 15 with symptoms, cardiovascular risk factors, or cognitive impairment) who accept treatment will be prescribed PAP therapy using whatever mode of pressure delivery the practitioner decides is clinically indicated. This could include autoPAP, bilevel PAP, adaptive servoventilation, and average volume assured pressure support. All three study sites provide home medical equipment and supplies through their sleep programs, and do not contract out this service. Patients will be scheduled for group or individual PAP set-up classes based on availability of classes and participant schedules. The type of PAP device prescribed will be left to the discretion of the clinician interpreting the sleep study. The mask interface will be selected at the preference of the patient, and no restrictions on type of mask used will be imposed. All equipment and supplies provided to participants are documented in the VA Computerized Patient Record System (CPRS). Patients will be informed of the use of wireless monitoring, how modems transmit data from their machines to manufacturer servers, and how VA clinicians access this data in accordance with national approvals for wireless monitoring. Patients are always provided an option to not participate in wireless monitoring through this shared decision-making process. In these cases, manual download of the device smartcard is required to obtain PAP compliance and efficacy data. Patients are provided with instructions on cleaning the equipment and masks, as well as instructions on basic trouble shooting of common problems related to PAP. We anticipate that 10% of participants who qualify for treatment with PAP therapy will decline initiation of therapy. These patients will be scheduled to meet with a sleep provider, based on usual clinical processes, to devise an alternate care plan or discharge back to primary care.

### Study procedures—follow-up

Ninety (90) days after each patient begins PAP therapy or other treatment, a follow-up visit will be scheduled with the care provider. This appointment will occur via in-person, telephone, or VTel (video telehealth) visits at the VA sleep center, community-based outpatient clinic (CBOC), or in the patient’s home. Participants will complete follow-up questionnaires at this time (see the Data Collection section for a list of study questionnaires). Sleep staff members who see patients in follow-up can change patient management strategies (e.g., mask type or mode of PAP therapy) based on their clinical judgement, and all changes will be documented using standardized templates in the VA CPRS. Patients struggling with PAP therapy may undergo as many telephone, video or additional in-person visits with a respiratory therapist or sleep technologist as necessary during the follow-up period to assist them with tolerating therapy; this is usual care at all of the participating VA sleep programs. After the 3-month visit, participants will be scheduled for routine sleep clinic follow-up visits as clinically indicated. It is anticipated that these will occur every 6–12 months after the initiation of therapy.

### Data collection—questionnaires

At the time of sleep testing, patients will fill out the following questionnaires:

1. A baseline questionnaire that collects demographic data and information about the patient’s experience with sleep apnea (see Additional file 1).
2. The Insomnia Severity Index (ISI) [21]
3. The Epworth Sleepiness Scale (ESS) [22]
4. The Functional Outcomes of Sleep Questionnaire (FOSQ-10) [23].

### Data collection—other measures

The following data will be collected:

1. Sleep test results, including AHI (3% and 4%), Oxygen Desaturation Index (3% and 4%), Time Spent (< 89% and < 90%), PSG Sleep Efficiency, and PSG Periodic Limb Movement Index.
2. PAP usage data, including Average Usage (Total Days and Days Used), Percentage of Days with Usage ≥ 4 h, and Residual AHI (events per hour).
  PAP treatment adherence and efficacy data will be obtained by daily transmission from wireless modems built into PAP units or via smartcard downloads. Use of wireless PAP monitoring in VHA is approved at the VA national level. Data will be stored on PAP device manufacturer websites (namely, EncoreAnywhere for Philips Respironics, or Airview for ResMed, Inc.) which VA sleep programs may access for provision of clinical care. Manual downloads of PAP data can be done during in-person assessments if the patient declines to use wireless technology.
3. Ninety (90) days after each patient begins PAP therapy or other treatment, a follow-up visit will be scheduled with the care provider. Patients will also fill out a follow-up questionnaire at that time (see Additional file 1), the ISI, ESS and FOSQ-10 questionnaires.

### Data collection for specific aim 3—negative predictive value of HSAT

Patients who undergo HSAT followed by PSG will provide data for Specific Aim 3 of the study when results of each test are compared. Also, patients who go to one of the study sites for in-lab PSG testing will be asked if they consent to participate in the research portion of this project which consists of adding 4 pieces of HSAT apparatus to the PSG set-up. This will enable simultaneous recording of HSAT and PSG, and will allow direct comparisons of test results from the same patient. The informed consent protocol for this portion of the study was approved by the VA Central IRB and by the R&D Committee at each site.

### Data storage and security

Electronic medical records generated from the study are stored in the VA CPRS and the VA Corporate Data Warehouse, which are secured and password protected. Additional study data will be entered into a system called VA REDCap (Research Electronic Data Capture) which is also secured and password protected. Research data will only be accessible to authorized personnel using password-protected and encrypted computers connected to VA secure networks. Paper records will be stored in locked cabinets within locked offices at each study site. Access to study data will be terminated for study personnel when they are no longer part of the research team. The Information Security Officer and Privacy Officer at each site will be notified within one hour of the improper use or disclosure of research data.

### Data analytic plan

Statistical models will be generated to determine whether patients in the DREAM pathway were evaluated and received treatment for OSA more quickly than those in the Traditional pathway (Specific Aim 1); to evaluate the claim that the DREAM pathway is as effective as the Traditional pathway for treating OSA patients (Specific Aim 2); and to test claims that HSAT approaches the efficacy of PSG in its ability to correctly identify OSA (Specific Aim 3). Further, based on the available data, additional exploratory analyses will be performed to predict HSAT results (Exploratory Analysis 1) and PAP adherence (Exploratory Analysis 2) via a machine learning modeling approach.

*Specific Aim 1* For the primary outcome, patients will be included for data analysis if they were referred for OSA and they completed sleep testing. An OLS (ordinary least squares) regression model will be composed to test the hypothesis that patients in the DREAM pathway were evaluated for OSA in fewer days (using the initial referral as the reference point) than those in the Traditional pathway, after controlling for demographics and covariates that indicated differences following distribution to treatment groups. Then, a follow up quantile regression analysis will be used to probe the effects at nine different percentiles ranging from 0.01 to 0.99. In contrast to OLS regression where the researcher examines the conditional mean of the distribution of the dependent variable, quantile regression allows us to examine the conditional median of the dependent variable at each of the poles in addition to points of interest between the poles. Thus, for example, if we want to examine the effect of pathway assignment (DREAM vs. Traditional) among patients who waited a greater number of days for testing (e.g., the 75th percentile of days waiting), only quantile regression could yield this effect. In this way, conclusions could be made for patients at varying levels of the dependent variable. For the secondary outcome, patients will be included for data analysis if they were referred for OSA, were diagnosed with OSA, and they initiated PAP treatment. An identical OLS/quantile regression approach will be taken to test the hypothesis that patients in the DREAM pathway would be initiated to PAP therapy in fewer days than the Traditional pathway.

**Fig. 1:**
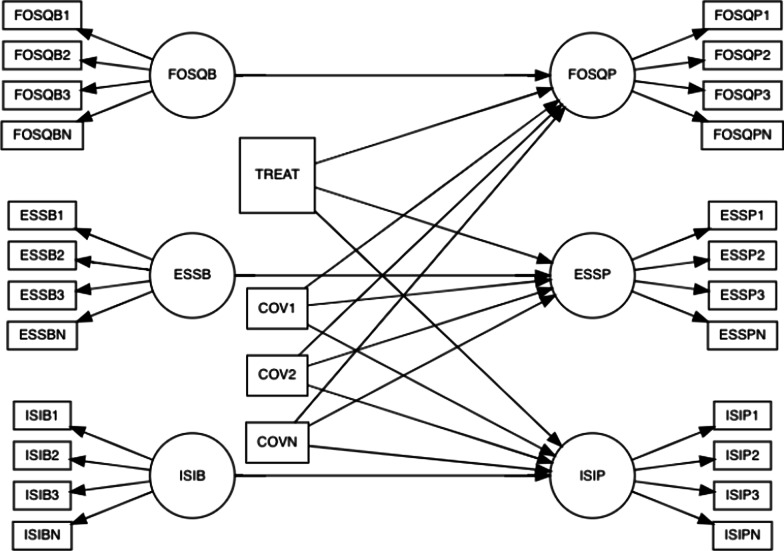
SEM template regressing FOSQ, ESS, and ISI on treatment, after controlling for baseline scores and covariates. Rectangles at sides of figure represent scale items; circles represent latent variables; rectangles in the middle of figure are covariates. Square in the middle of the figure is the treatment variable. Label including/ending in “B” indicates baseline measure; Label including/ending in P indicates post-PAP measure. SEM = structural equation modeling; FOSQ = functional outcomes of sleep questionnaire (FOSQ-10); ESS = Epworth sleepiness scale; ISI = Insomnia severity index. COV = covariate. TREAT = treatment group (0 = traditional; 1 = DREAM)

*Specific Aim 2* For the primary and secondary outcomes, the goal is to test the hypothesis that the DREAM pathway is equal in its efficacy to the Traditional pathway as pertains to the adherence to OSA treatment and to outcomes related to the quality of OSA treatment. Whereas a non-inferiority approach (i.e., frequentist statistics) is often used in these cases to test whether an experimental group was not worse than a control group (i.e. assumption of the null hypothesis), there are drawbacks to this approach including additional degrees of freedom related to selecting the threshold by which a difference would be considered meaningful; that conclusions related to this approach are resigned to making inferences based on single point estimates; and that the interpretation of non-inferiority test results can be difficult to interpret and misleading [24]. In contrast, Bayesian modeling can provide more comprehensive and unambiguous results by indicating, (1) intervals including a range of plausible coefficients, and (2) explicit numerical evidence supporting the null hypothesis in the form of Bayes factors (BF). In Bayesian statistics, a distribution of the effect is produced such that the researcher can visualize a range of the effect of the pathway on the dependent variable and the likelihood that each potential estimate is to occur. A 95% range of estimates (known as the credible interval) that includes zero would indicate the range of plausible values for the DREAM pathway overlaps with those for the Traditional pathway and therefore the DREAM pathway would be considered to not perform worse. The BF statistic provides a ratio of the likelihood of the null hypothesis to the alternative hypothesis such that a Bayes factor between 1 and 3 is inconsequential, BF between 3 and 20 is positive, and BF between 20 and 150 is strong [25] (see van Ravenzwaaji [24] for more details on computing BF). Thus, for the primary outcome, a credible interval and BF will be computed to indicate the likelihood of the null hypothesis that the level of PAP adherence (i.e., the average number of hours that PAP therapy is used per night) among patients diagnosed with OSA does not differ for patients in the DREAM and Traditional pathways. In both outcomes, demographic variables and covariates that indicate differences following distribution to treatment groups will be entered as statistical controls. For the secondary outcome, FOSQ-10, ESS, and ISI scores will replace PAP adherence as the dependent variable, and Bayesian structural equation modeling (SEM) will be implemented to accommodate the latent structure of the questionnaires (i.e., questionnaire items specified onto unobserved factors; see Fig. 1 for model specification). Initially, confirmatory factor analyses (CFA) will be performed to select the best from a series of competing models (one-factor, correlated factor, and bifactor) to establish the optimal structure and relations of the questionnaires. Then paths from the established factor structure from the CFA will be regressed onto the treatment pathway, after controlling for demographic and other relevant covariates. From the resulting coefficient, the credible interval and BF will be computed to test the null hypothesis that the groups did not differ on changes in sleep quality (FOSQ-10), levels of sleepiness (ESS), and insomnia (ISI) that occur following treatment.

For both outcomes of Aim 2, patients will be included in the analyses if they completed the baseline questionnaires, were diagnosed with OSA, and engaged in therapy. If the patient met these requirements but failed to complete the post-treatment questionnaires, attempts will be made to contact the patient to retrieve this data. Data that are unable to be retrieved will be estimated with multiple imputation techniques.

*Specific Aim 3* The negative predictive value (NPV) of HSAT will be examined for all patients who undergo both HSAT and PSG testing for sleep apnea. Using the PSG as the gold standard for OSA testing, NPV is computed by dividing the number of patients who tested negative on HSAT by the sum of patients who tested negative on the HSAT plus patients who tested negative on HSAT but tested positive for OSA on PSG. Based on our estimate of collecting 10,000 sleep studies during the project period, and results from preliminary data indicating that 15% of HSATs are negative, approximately 1500 patients will undergo both HSAT and PSG and be subjected to analysis. Further, subgroup computations will be made to determine whether there are any differences on NPV for various comorbidities (COPD, diabetes, heart disease, hypertension, mood disorders, PTSD) and for high-risk patients (as compared to low risk for OSA).

### Exploratory analyses

In the process of obtaining sufficient numbers of home sleep tests to calculate the NPV, we will also have a large dataset of positive HSAT results. We will conduct exploratory analyses to evaluate patient characteristics (including comorbidities, body mass index [BMI], gender, etc.) that predict a positive HSAT result. Following the examination of specific aims relevant to the hypotheses of this project, the available data will allow us to compose additional statistical models building on the study results. These analyses will enable us to generate recommendations regarding the use of HSAT for at-risk patient populations.

## Discussion

### Expected outcomes

We hypothesize that the DREAM clinical pathway will result in shorter wait times from time of referral to the date of diagnostic testing and initiation of treatment for OSA when compared to initial in-person consultation (Traditional pathway). We also hypothesize that PAP adherence in the DREAM condition will not be inferior to initial in-person consultation; patient-reported outcomes and satisfaction will not be inferior in the DREAM versus the Traditional pathway. Data collected in this study will enable identification of sub-groups of Veteran patients most likely to benefit from the DREAM clinical pathway or home sleep apnea testing (HSAT). HSAT results from at-risk patients will help to inform clinicians and policymakers regarding usage guidelines for at-home OSA testing. Finally, results from Aim 3 of this study will enable us to calculate a definitive NPV for HSAT.

### Study limitations and challenges

One concern is the potential variation in clinician preference for PSG vs. HSAT at each study site. The study design will enable us to accommodate individual clinician preferences related to PSG or HSAT by adding HSAT equipment to PSG set-ups when patients consent to allow it. Thus, data from home devices will simultaneously be captured during in-lab testing. This approach is adaptable to shifting referral patterns and clinician preferences for PSG or HSAT which are anticipated to change over the study period. A second concern is the ability to capture polysomnography on all or most negative HSAT studies. Due to the pragmatic nature of the study, we anticipate that not all patients will agree to have a confirmatory polysomnogram, nor will their clinicians always decide that a follow-up PSG is indicated. However, we believe that a sufficient amount of data will be collected to ensure definitive outcomes. A third concern is the effect of the COVID-19 pandemic on study results. At all three study sites, sleep testing was suspended and other clinical services were limited for several months in 2020. More recently, sleep services were restored at the study sites, but surges in COVID-19 cases could cause additional disruptions in the future. Also, the announcement by Philips Respironics in June 2021 that they were recalling most of their PAP devices produced since 2008 has resulted in a massive world-wide shortage of PAP devices. Consequently, Veterans diagnosed with sleep apnea are being tried on alternative therapies such as oral mandibular advancement devices (OMAD), positional therapy, or supplemental oxygen with only the very sickest patients and most severe cases of OSA receiving PAP therapy. As this shortage of PAP devices abates, we anticipate that some Veterans in the moderate and mild category of sleep apnea who were given alternative treatments may benefit from PAP therapy. In any case, results from this study will reflect VA sleep medicine practices over the course of this tumultuous period. Another challenge for this study stems from its multi-site design and non-standardized clinical practices/preferences at each of the participating VA sleep programs. While our statistical models will strive to identify and account for differences among the study sites, we believe that the results will reflect real-world variations in clinical practice and will thus be applicable to a broad spectrum of sleep medicine programs.

### Dissemination of study results

Study results will be disseminated through a variety of methods, including, (1) publication of manuscripts summarizing findings; (2) presentation of results at national society meetings (American Thoracic Society, American College of Chest Physicians, Associated Professional Sleep Society); (3) presentation of results via national sleep medicine networks. Results of Aims 1 and 2 should be available within the first 3–4 years of study activation. Results from Aim 3 will be available at intervals depending on the total number of Veterans undergoing sleep testing at all study sites, and could be presented incrementally based on interval analysis.

## Supplementary Information

**Additional file 1.** Study Questionnaires.

## Data Availability

The datasets generated during and/or analysed during the current study will be available from the corresponding author on reasonable request.

## Abbreviations

AASM: American Academy of Sleep Medicine
AHI: Apnea–hypopnea index
BF: Bayes factor
BMI: Body mass index
CA: California
CBOC: Community-based outpatient clinic
CFA: Confirmatory factor analysis
COPD: Chronic obstructive pulmonary disease
COV: Covariate
COVID: Coronavirus disease
CPRS: Computerized patient record system
DREAM: Direct Referral for Apnea Monitoring
ESS: Epworth Sleepiness Scale
FOSQ: Functional Outcomes of Sleep Questionnaire
HSAT: Home sleep apnea testing
HSR&D: Health Services Research & Development
IRB: Institutional Review Board
ISI: Insomnia Severity Index
NPV: Negative predictive value
OLS: Ordinary least squares
OMAD: Oral mandibular advancement device
OR: Oregon
OSA: Obstructive sleep apnea
PA: Pennsylvania
PAP: Positive airway pressure
PSG: Polysomnography
PTSD: Post-traumatic stress disorder
R&D: Research & Development
REDCap: Research Electronic Data Capture
SEM: Structural equation modeling
VA: Veterans Affairs
VHA: Veterans Health Administration
VTel: Video telehealth
