# Supplementary material for: Study protocol to assess de-implementation of the initial provider encounter for diagnosis and treatment of obstructive sleep apnea: the DREAM (Direct Referral for Apnea Monitoring) Project
Source: BMC Pulm Med. 2022 Apr 2;22:123. doi: 10.1186/s12890-022-01899-y (PMC8976272; doi:10.1186/s12890-022-01899-y)
Supplement: Supplementary file 1 — Additional file 1. Study Questionnaires. [file 12890_2022_1899_MOESM1_ESM.docx]

Appendix 1. Baseline Questionnaire

Last 4

Name: ___________________________ Digits SS# ________ Date: _________

**Please respond to the following questions by marking your answers with an “X”**

**YES NO**

1. **Are you of Hispanic, Latino, or Spanish Origin?** ⬜ ⬜

If **YES**:  Are you ⬜ Mexican, Mexican-American, Chicano

                ⬜ Puerto Rican

⬜ Cuban

⬜ Other Hispanic (Salvadoran, Dominican, Colombian,

Guatemalan, Spaniard, Ecuadorian, etc.)

⬜ Prefer not to answer

1. **What is your race?**

⬜ American Indian or Alaska Native

⬜ Asian or Pacific Islander

⬜ Black / African-American

⬜ White / Caucasian

⬜ Other: _____________________________

⬜ Prefer not to answer

1. **What is your current marital status**?

⬜ Married, living with spouse

⬜ Married, separated

⬜ Widowed

⬜ Divorced

⬜ Never married

1. **Are you currently employed**?

⬜ YES, Employed full time

⬜ YES, Employed part-time or on-call

⬜ NO, Retired

⬜ NO, Looking for employment

⬜ NO, Unemployed because of health

⬜ NO, Other reason: _______________________________

**5. What is the highest grade or level of school that you completed?**

⬜ 8^th^ grade or less

⬜ Some high school, but did not graduate

⬜ High school graduate or GED

⬜ Some college or 2-year degree

⬜ 4-year college graduate

⬜ More than a 4-year college degree

|  | **Never** | **1 day or less a week** | **2-3 days a week** | **4-6 days a week** | **7 days a week** |
| --- | --- | --- | --- | --- | --- |
| 1. **About how often do you use technology like smart phones, tablets, computers, websites, apps, or the internet?** | ⬜ | ⬜ | ⬜ | ⬜ | ⬜ |

| 1. **Have you used any of the following resources to learn more about your sleep? (*select all that apply*)** |
| --- |

- Books or magazines
- Brochures and informational material
- Internet YouTube videos or websites
- Social media like Facebook or Twitter
- Mobile apps that track your sleep on your smart phone

Wearables like Fitbit or Oura

ring for sleep

- CPAP mobile apps like myAir™ or DreamMapper
- REVAMP VA App – the Remote Veteran Apnea Management Platform
- Other _____________________
- None

|  | ***None Some A Lot*** | | | | | | | | | | |
| --- | --- | --- | --- | --- | --- | --- | --- | --- | --- | --- | --- |
| 1. **How much do you understand about sleep apnea and how it can affect your health?** | *0* | *1* | *2* | *3* | *4* | *5* | *6* | *7* | *8* | *9* | *10* |

| 1. **If you your sleep test shows you have sleep apnea, are you willing to use a CPAP device**   **(a mask you wear at night that helps you to breathe)?** | Definitely  No  ⬜ | No  ⬜ | Maybe  ⬜ | Yes  ⬜ | Definitely Yes  ⬜ |
| --- | --- | --- | --- | --- | --- |
| 1. **How satisfied are you with how long it took to get a sleep test?** | Extremely Dissatisfied  ⬜ | Dissatisfied  ⬜ | Neutral  ⬜ | Satisfied  ⬜ | Extremely Satisfied  ⬜ |
| 1. **Overall, how satisfied are you with your sleep care from the VA so far?** | Extremely Dissatisfied  ⬜ | Dissatisfied  ⬜ | Neutral  ⬜ | Satisfied  ⬜ | Extremely Satisfied  ⬜ |

| 1. **If you would like to share any additional comments about your sleep test or sleep care at the VA, please write them below.** |
| --- |

***Thank you for completing the Baseline survey. Your answers are important.***

***We will contact you 3-months after treatment starts for a Follow-up survey.***

Appendix 2. Follow-up Questionnaire

Last 4

Name: ________________________________ Digits SS# ________ Date: _________

**Please respond to the following questions by marking your answers with an “X”**

|  | **Yes** | **No** | **Unsure** |
| --- | --- | --- | --- |
| 1. **Have you received the results of your sleep test?** | ⬜ | ⬜ | ⬜ |
| 1. **Did your sleep test say you have sleep apnea?** | ⬜ | ⬜ | ⬜ |

| 1. **What treatment was recommended for your sleep apnea? (*select all that apply*)** |
| --- |

- CPAP or BPAP (a mask you wear at night

that helps you to breathe)

- Weight Loss
- Change my sleeping position

| 1. **Have you used any of the following resources to learn more about your sleep? *(select all that apply)*** |
| --- |

- Other treatment:

- None / Not sure
- I don’t have sleep apnea
- Books or magazines
- Brochures and informational material
- Internet YouTube videos or websites
- Social media like Facebook or Twitter
- Mobile apps that track your sleep on your smart phone

Wearables like Fitbit or Oura ring

CPAP mobile apps like myAir™ or DreamMapper

- REVAMP VA App – the Remote Veteran Apnea Management Platform
- Other _____________________
- None

|  | | | ***None Some A Lot*** | | | | | | | | | | | | | | | | | | |  |
| --- | --- | --- | --- | --- | --- | --- | --- | --- | --- | --- | --- | --- | --- | --- | --- | --- | --- | --- | --- | --- | --- | --- |
| 1. **How much do you understand about sleep apnea and how it can affect your health?** | | | *0* | | *1* | *2* | *3* | | *4* | | *5* | | *6* | | *7* | | *8* | | *9* | | *10* |  |
|  | ***Not***  ***at all Somewhat Extremely*** | | | | | | | | | | | | | | | | | | | | | |
| 1. **How confident are you that you can manage your sleep problems?** | *0* | *1* | | *2* | | *3* | | *4* | | *5* | | *6* | | *7* | | *8* | | *9* | | *10* | | |

| 1. **Overall, how satisfied are you with your sleep care from the VA so far?** | **Extremely Dissatisfied**  ⬜ | **Dissatisfied**  ⬜ | **Neutral**  ⬜ | **Satisfied**  ⬜ | **Extremely Satisfied**  ⬜ |
| --- | --- | --- | --- | --- | --- |

|  | **Never** | **1-2 nights a week** | **3-4 nights a week** | **5-6 nights a week** | **Every night** | **I was not given CPAP or BPAP** |
| --- | --- | --- | --- | --- | --- | --- |
| 1. **How often are you using your CPAP or BPAP?** | ⬜ | ⬜ | ⬜ | ⬜ | ⬜ | ⬜ |

|  | **Less than 1 hour** | **1-2 hours** | **3-4 hours** | **5-6 hours** | **7 or more hours** | **I was not given CPAP or BPAP** |
| --- | --- | --- | --- | --- | --- | --- |
| 1. **When you do use your CPAP or BPAP, how many hours do you use it per night?** | ⬜ | ⬜ | ⬜ | ⬜ | ⬜ | ⬜ |

| 1. **If you would like to share any additional comments about your sleep test or sleep care at the VA, please add them below.** |
| --- |
|  |

***Thank you for completing the Follow-up survey. Your answers are important and will***

***help us to improve care for Veterans.***
